# Articulation rehabilitation induces cortical plasticity in adults with non-syndromic cleft lip and palate

**DOI:** 10.18632/aging.103402

**Published:** 2020-07-03

**Authors:** Zhen Li, Wenjing Zhang, Chunlin Li, Mengyue Wang, Songjian Wang, Renji Chen, Xu Zhang

**Affiliations:** 1 School of Biomedical Engineering, Capital Medical University, Beijing, China; 2 Beijing Key Laboratory of Fundamental Research on Biomechanics in Clinical Application, Capital Medical University, Beijing, China; 3 Department of Oral and Maxillofacial Plastic and Trauma Surgery, Center of Cleft Lip and Palate Treatment, Beijing Stomatological Hospital, Capital Medical University, Beijing, China

**Keywords:** non-syndromic cleft lip and palate (NSCLP), articulation rehabilitation (AR), gray matter volume (GMV), cortical thickness (CT), cortical complexity

## Abstract

In this study, we investigated brain morphological changes in adults with non-syndromic cleft lip and palate (NSCLP) after articulation rehabilitation (AR). High-resolution T1 weighted brain magnetic resonance imaging data were analyzed from 45 adults with NSCLP after palatoplasty: 24 subjects were assessed before AR (bNSCLP) and 21 subjects were assessed after AR (aNSCLP). In addition, there were 24 age and sex matched controls. Intergroup differences of grey matter volume were evaluated as a comprehensive measure of the cortex; cortical thickness and cortical complexity (gyrification and fractal dimensions) were also analyzed. As compared to controls, the bNSCLP subjects exhibited altered indexes in frontal, temporal, and parietal lobes; these morphological changes are characteristic for adults with NSCLP. Importantly, as compared to the bNSCLP and control subjects, the aNSCLP subjects exhibited cortical plasticity in the regions involved in language, auditory, pronunciation planning, and execution functions. The AR-mediated cortical plasticity in aNSCLP subjects may be caused by AR-induced cortical neurogenesis, which might reflect the underlying neural mechanism during AR.

## INTRODUCTION

Non-syndromic cleft lip and/or palate (NSCL/P) is one of the most common maxillofacial deformities. Its effect on speech, hearing, appearance, and cognition can lead to long-lasting adverse outcomes [1–3]. Even after surgery, many adults have a ‘CLP speech’, which is accompanied by hypernasality and/or nasal emission. To improve articulation, they often require speech therapy or articulation rehabilitation (AR). The development of the face and the brain are closely related under both normal and pathologic conditions [4–6]. A recent study has indicated a unified developmental basis for orofacial clefting and disrupted cortical interneurons development in a mouse model [5]. Several studies have shown a correlation between abnormal brain structure changes and neurobehavior disorder in NSCL/P cohorts [7–11]. Brain morphological analyses of NSCL/P adults have indicated brain changes, such as increased frontal lobe volumes, decreased temporal and occipital lobe volumes, lower cerebellum volumes, and higher rates of cavum septum pellucidum compared with normal controls [11–14]. A pediatric NSCL/P study has found that compared with age-matched controls, children with NSCL/P (6 to 14 years of age) have increased global cortical gray matter volumes (GMV) with decreased volumes of subcortical grey matter and cerebral white matter structures [8]. Besides, they have increased cortical thickness (CT) in left frontal and right parietal lobes and decreased CT in right superior frontal cortex [8]. However, to our knowledge, it is not known whether AR might affect the brain morphological changes.

Brain morphological analysis is a stable and reliable method to investigate the brain structures [15, 16]. Total intracranial volume (TIV) and gray matter volume (GMV) are computed as the number of voxels belonging to gray matter with and without white matter segmentations, multiplied by voxel volume. Cortical thickness is defined as the Euclidian distance between the inner and outer cortical surfaces [17]. Gyrification refers to the process of cortical folding [18]. We have previously used a surface-based approach by computation of absolute mean curvature (AMC) as an estimation of cortical folding [19]. AMC corresponds to the local amount of gyrification; it can be considered a measure of the sharpness of gyri and sulci [19]. Fractal dimension (FD) is the slope of the log of box number versus the log of inverse of box size; an increase in the FD value indicates an increase in complexity [20]. Here we used spherical harmonic (SPH) reconstructions to extract the surface complexity information [21].

In this study, we have investigated the brain morphological changes before and after articulation rehabilitation (AR) in adults with non-syndromic cleft lip and palate (NSCLP). We have analyzed their GMV intergroup differences as a comprehensive measure of the cortex, and their CT and cortical complexity to evaluate the thickness and variation of cortical folding. We hypothesized that adults with NSCLP might have brain morphological changes that might partially underlie their behavioral and cognitive deficits, and that AR might induce cortical plasticity in brain regions involved in language and auditory functions.

## RESULTS AND DISCUSSION

### Demographic data

In this study, we analyzed high-resolution T1 weighted brain magnetic resonance imaging data obtained from 45 adults with NSCLP after palatoplasty: 24 subjects were before AR (bNSCLP group) and 21 subjects were after AR (aNSCLP group). In addition, 24 subjects were age and sex matched controls. There were no statistical differences in age, gender, educational background, and total intracranial volume (TIV) among the groups. Articulation test scores were greatly improved in the aNSCLP group (Table 1).

**Table 1:** Demographic and clinical data of the subjects.

	**bNSCLP (24)**	**aNSCLP (21)**	**NC (24)**	***P***
Age (years)	23.00(21.25-27.50)	24.00(19.00-25.75)	22.00(21.00-23.00)	0.581 c
Gender (M/F)	12/12	14/7	15/9	0.488 a
Education (years)	13.50(12.00-15.00)	15.00(12.25-15.00)	14.00(14.00-15.00)	0.446 c
ATS	47(13-92)	94(86-100)	---	<0.001 b
TIV	1374.89(1313.39-1460.20)	1347.81(1294.53-1434.40)	1472.12(1073.46-1741.15)	0.147 c

Non-normally are presented as median (Interquartile range, IQR). *P*<0.05 indicates statistically significant.

a The Chi-square test for dichotomous data.

b The Mann-Whitney U-test for non-normally distributed data between two groups.

c The Kruskal-Wallis Test for non-normally distributed data among three groups.

TIV: Total intracranial volume

ATS: Articulation test score

### Morphological changes in NSCLP subjects compared with controls

**Figure 1:**
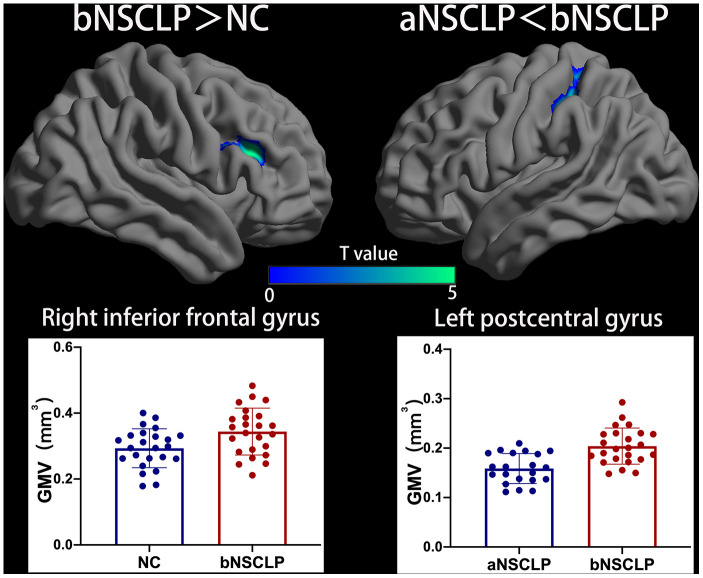
**Increased gray matter volume (GMV) in bNSCLP compared with aNSCLP and controls.** Voxel-based analysis showed that the bNSCLP group exhibited increased GMV in right IFG compared with controls. Besides, bNSCLP group had increased GMV in left PoCG compared with aNSCLP (*P*<0.05, cluster-level FWE-corrected). Color bars indicate T-values. The 3D brain maps show the spatial location of intergroup different regions, and the boxplots below show the GMV values for the different groups. PoCG: postcentral gyrus; IFG: inferior frontal gyrus.

**Figure 2:**
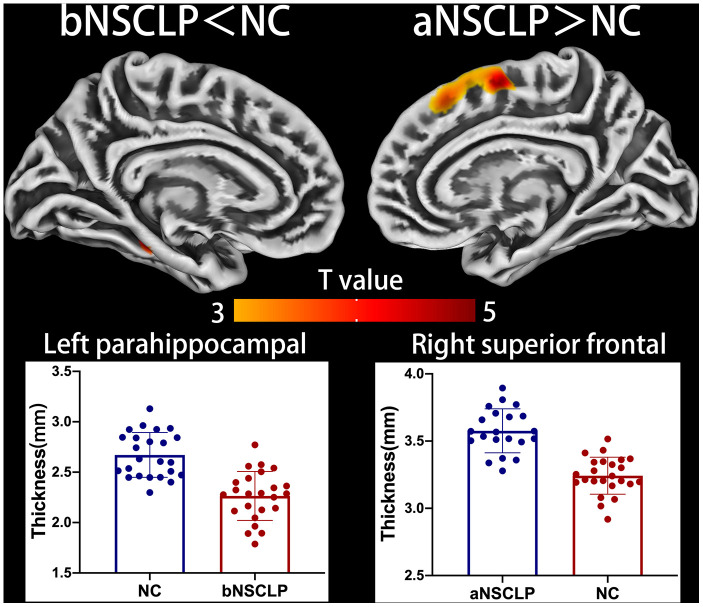
**Altered cortical thickness between every pair in the three groups.** Regions with intergroup differences are shown on lateral or medial views. Two-sample t-test for every pair in the three groups was used; statistical significance, *P*<0.05, FWE-corrected. Color bars indicate T-values. The 3D brain maps show the spatial location of intergroup different regions, and the boxplots below show surface index values for the groups.

Using voxel-based analysis, the bNSCLP group exhibited increased GMV in right inferior frontal gyrus (IFG) compared with controls (*P*<0.05, cluster-level FWE-corrected, Figure 1, Supplementary Table 1). In addition, the bNSCLP group had decreased CT in left parahippocampal (PHIP) and fusiform compared with the controls (*P*<0.05, peak-level FWE-corrected, Figure 2, Supplementary Table 2).

**Figure 3:**
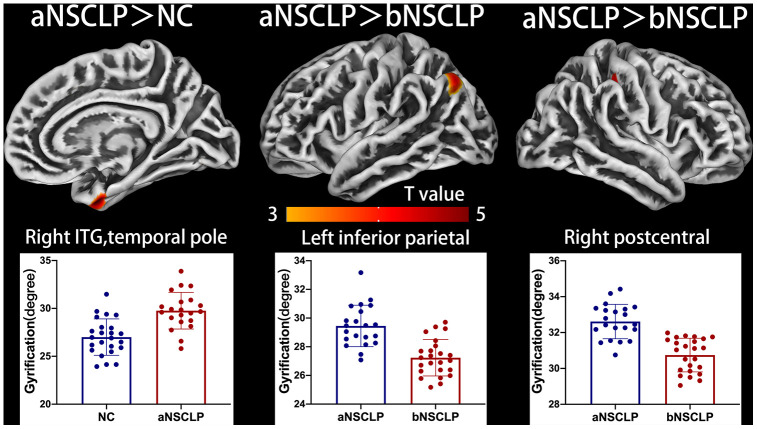
**Altered gyrification between every pair in the three groups.** Regions with intergroup differences are shown on lateral or medial views. Two-sample t-test for every pair in the three groups was used; statistical significance was *P*<0.05, FWE-corrected. Color bars indicate T-values. The 3D brain maps show the spatial location of intergroup different regions, and the boxplots below show surface index values for different groups.

**Figure 4:**
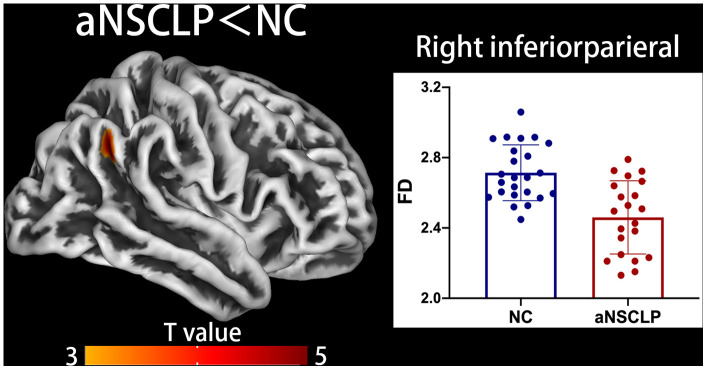
**Decreased fractal dimension (FD) in aNSCLP compared with controls.** Regions with intergroup differences are shown on lateral views. Two-sample t-test for every pair in the groups was used; statistical significance was *P*<0.05, FWE-corrected. Color bars indicate T-values. The 3D brain map shows the spatial location of intergroup different regions, and the boxplot below shows the surface index values for the two groups.

In the aNSCLP group, CT was significantly increased in right superior frontal gyrus (SFG) compared with controls (*P*<0.05, peak-level FWE-corrected, Figure 2, Supplementary Table 2). Using surface complexity analysis, gyrification was increased in right temporal pole and inferior temporal gyrus (ITG, *P*<0.05, peak-level FWE-corrected, Figure 3, Supplementary Table 2). FD was decreased in right inferior parietal lobule (IPL, *P*<0.05, peak-level FWE-corrected, Figure 4, Supplementary Table 2).

### Morphological changes in NSCLP subjects after AR

Importantly, the aNSCLP group had a decreased GMV in left postcentral gyrus (PoCG) compared with the bNSCLP group (*P*<0.05, cluster-level FWE-corrected, Figure 1, Supplementary Table 1). In addition, the aNSCLP group showed increased gyrification in left IPL (*P*<0.05, peak-level FWE-corrected), right PoCG and right supramarginal gyrus (SMG) compared with the bNSCLP group (*P*<0.05, peak-level FWE-corrected, Figure 3, Supplementary Table 2).

The brain morphological changes in the bNSCLP group were characteristic for the NSCLP cohorts. Compared with bNSCLP and control groups, the aNSCLP group had cortical plasticity in the regions involved in language functions (left IPL, right SMG), auditory functions (right temporal pole and right ITG), pronunciation planning and execution functions (right SMA), which might reflect the underlying neural mechanisms during AR.

### Brain morphologic changes of adults with NSCLP

### Increased GMV in right inferior frontal gyrus (IFG)

In line with previous GMV analyses [12, 14, 22], we found an increased GMV in right inferior frontal gyrus (IFG) in the bNSCLP group compared with the control group. This may represent the characteristic brain changes in the NSCLP cohorts. IFG belongs to ventral frontal cortex, which is involved in the attention network. The human brain features dorsal and ventral attention networks, which have been linked to mediating orienting towards a stimulus based on behavioral significance [23] and detection of salient targets, particularly in unattended locations [24, 25]. The NSCLP cohorts have been shown to exhibit elevated levels of attention dysfunction [1, 3, 9, 10, 26]. Thus, the GMV increase in right IFG in the bNSCLP group might represent a functional compensation for attention deficit in this population.

The right IFG is involved in semantic and syntactic processing. Although the left hemisphere is dominant in language processing, the right hemisphere has been also associated with the language processing. For example, semantic unification operations are under top-down control of the left, and in the case of a discourse, also the right inferior frontal cortex [27]. In a meta-analysis study that compared left and right hemisphere activities related to language processing (phonological, lexico-semantic, and sentence or text processing), the activation peaks in the right hemisphere represent about one third of the activation peaks in the left hemisphere [28]. Moreover, in most cases, the right hemisphere activations were in the homotopic areas, suggesting a strong interhemispheric influence [28]. Thus, the GMV increase in the right IFG in the bNSCLP subjects might represent a functional compensation for their impaired language function.

### Increased GMV in left postcentral gyrus (PoCG)

Compared with control (PoCG; P<0.001, uncorrected) and aNSCLP groups (P<0.05, cluster-level FWEcorrected), the bNSCLP group had an increased GMV in left postcentral gyrus. A previous study has demonstrated increased GMV in bilateral PoCG in children with NSCLP [8], indicating that this is an important region in the NSCLP cohorts. A previous analysis of stuttering adults has found increased GMV in the precentral and postcentral gyrus, inferior parietal, and temporal lobule of bilateral hemisphere, which might represent a long-term functional compensation for the cerebella and medulla function deficiency [29]. Functional MRI studies have revealed that left PoCG is involved in the phonological brain network, which is responsible for effective phonological processing. It encompasses mostly left hemisphere areas like the inferior parietal lobule (including supramarginal and angular gyri), inferior frontal cortex, postcentral and precentral gyri, superior and middle temporal gyri, and fusiform and dorsolateral prefrontal cortex [30, 31]. Brain damage to left precentral and postcentral gyri could lead to apraxia of speech [32, 33], and damage to inferior parietal (supramarginal gyrus) and somatosensory (postcentral gyrus) regions could lead to phonological errors during single-word production (picture naming) in post-stroke aphasia [34–36]. The increased GMV in left PoCG in bNSCLP adults may be the result of a long-term functional compensation for their impaired language function. During articulation rehabilitation, the function of the whole brain language processing neural network is enhanced, resulting in the weakened functional compensation of the left PoCG.

### Decreased CT in left parahippocampal gyrus (PHIP)

In contrast to the surface-based analysis in NSCL/P children [8], we found no regions with increased CT in the bNSCLP adult group compared with controls. We think this may be due to the different brain maturity in children vs adults. In addition, the bNSCLP group exhibited a decreased CT in left PHIP, a region closes to the statistically higher activation area in NSCLP population in our previous Chinese subvocalization task study [37]. Hippocampus (HIP) and PHIP are both deep brain areas important to memory. The PHIP receives input from all areas of the cerebral cortex, processing several types of sensory information [38, 39].

NSCLP cohorts were shown to have an increased incidence of neurobehavioral problems including learning disability, impaired language function, psychosocial adjustment issues, attentional deficit, and persistently reduced academic achievement [1–3, 9, 40, 41]. The morphological changes in bNSCLP adults found in our study, including the altered structures involved in attention network (right IFG), brain language macrocircuits (right IFG, left PoCG), and working-memory structures (HIP and PHIP), may represent the underlying neural mechanisms for these functional deficits.

### Brain morphologic changes during AR

### Increased gyrification in parietal, frontal, and temporal lobes

Our study showed that compared with the bNSCLP group, the aNSCLP group had an increased gyrification in left inferior parietal lobule (IPL), the junction part of right PoCG, and right SMG. The left IPL was activated in syntactic processing task, with the left precuneus and the right posterior MTG [27]. Besides, as mentioned above, it was involved in semantic processing network, which seems to include at least left IFG, left superior/middle temporal cortex, and (left) inferior parietal cortex [27].

In addition, the aNSCLP group had significantly increased gyrification in right temporal pole and right inferior temporal gyrus (ITG) (Figure 3, Supplementary Table 2) compared with the control subjects. Since ITG belongs to the auditory cortex, which provides an important input for speech. We speculate that regions with increased gyrification were functionally recruited during AR, their function was greatly improved.

### Increased CT in right superior frontal gyrus (SFG)

After AR, the aNSCLP group had a significantly increased CT in the right superior frontal gyrus (SFG), especially in the supplementary motor area (SMA), compared with the controls (Figure 2, Supplementary Table 2). The SMA is involved in motor planning and motor execution. An abnormal activation in SMA and anterior insula was found in stutters [42]. Alario et al have found that the SMA is involved in the motor movement important for articulation [43]. Therefore, we speculate that the increase in CT of SMA in the aNSCLP group is related to the vocalization exercises in this group.

### Decreased FD in right IPG

After rehabilitation training, compared with controls, the aNSCLP subjects exhibited decreased FD values in right IPG (Figure 4, Supplementary Table 2). The right IPG is involved in non-spatial attributes, such as reorienting of attention, arousal, and target detection [44, 45]. The impact of repetitive transcranial magnetic stimulation to the right IPL may indirectly affect dorsal network function, as has been suggested to occur in hemineglect [44, 45]. Event-related potentials studies indicated that asymmetric effect of attended location on gradients might be related to neglect of the left hemispace after right parietal injury, suggesting the role of the right IPL in modulating frontal lobe attention network activity [46].

Regions with high FD values generally appear to be more periodically spaced (e.g., like a sine wave with regular peaks and troughs). This may be because periodically spaced structures also tend to fill more space over the range of scales examined for derivation of complexity values [21]. A positive correlation was shown between whole-brain FD, IQ, and other cognitive test scores, suggesting a link between higher cortical complexity and higher functioning [47–49]. Regarding the decreased FD in right IPG in the aNSCLP group compared with controls, we speculate that the right IPL was not functionally involved in the training process. AR focused on improving articulation clarity, which may not be regulated by the right IPL.

Compared with bNSCLP and normal controls, the aNSCLP group had widespread altered morphologic changes in brain regions involved in language functions (left IPL and right SMG), auditory functions (right temporal pole and right ITG), and pronunciation planning and execution (right SMA). Adults with NSCLP in our study underwent the AR training for three months or more, until their articulation test scores reached 86 points. During the rehabilitation process, they had to learn and understand the method of AR, imitate the instructor, and vocalize repeatedly, to achieve a nearly normal vocalization. During this process, the brain regions involved in language, auditory, and pronunciation functions were intensely involved. These regions likely represent the brain functional networks required for improvement of speech and auditory functions in the NSCLP population.

### AR-induced neurogenesis in NSCLP subjects

Recent studies have shown that after short-term training (skill learning, memory training, etc.), the cortex undergoes remodeling, which is mainly manifested by gray matter changes and white matter structure remodeling [50, 51]. Neurogenesis, synapse generation, and vascular adaptation changes all contribute to gray matter changes [50, 51]. We hypothesize that the significantly enhanced surface indexes (CT and gyrification) of brain regions in the aNSCLP subjects are due to their improved function during the intensive training process, which may lead to increased neurogenesis. Adult neurogenesis is the formation of functional, mature neurons from neural stem cells in specific brain regions, where new neurons are generated throughout life and integrated into established neuronal circuits. Neurogenesis can lead to increase in GMV, cortical thickness and cortical folding rate (gyrification and FD). Articulation training can cause remodeling of white matter structure and changes in cortex. For example, high axon tension can lead to an increase in cortex folding [52, 53]. However, in our study, we found no GMV increase in the aNSCLP group. GMV is a comprehensive response of the cortex; several surface indexes (CT, gyrification, and FD) are related to its variation, which might reduce its sensitivity to short-term changes. Instead, surface indexes may be more sensitive as each of them represents one particular feature. This highlights the importance to analyze the cortical changes in combination with different indexes for a comprehensive evaluation of the cortical morphology.

### Possible gene expression changes in NSCLP subjects

Multiple genes contribute to molecular pathogenesis for facial development of NSCLP, including *IRF6, VAX1*, and *MAFB* [6, 54]. In addition to studies demonstrating that face and brain development are closely related [4–6], a mouse study has revealed a unified developmental basis for orofacial clefting and disrupted cortical interneuron development [5]. The characteristic brain changes in the bNSCLP group are likely due to the NSCLP-associated genes. Differential gene expression has been shown to lead to brain structure changes, such as altered gyrification [52, 53]. We searched for the expression of *IRF6, VAX1*, and *MAFB* using the Allen Human Brain Atlas (AHBA) [55] and found an increased *MAFB* expression in left PoCG and right IFG (regions with increased GMV in bNSCLP) compared with other regions. These data suggest that MAFB might be responsible for the characteristic brain morphologic changes in the bNSCLP cohorts, and might regulate not only facial anomalies, but also disruptions in central nervous system development, as indicated in animal studies [5].

## MATERIALS AND METHODS

### Subjects

This study recruited 24 adults with NSCLP after palatoplasty without AR (bNSCLP group), 21 adults with NSCLP after palatoplasty and after AR (aNSCLP group), and 24 age and sex matched controls (NC group) in Beijing Stomatological Hospital affiliated to Capital Medical University, from April 2016 to November 2016. There were no significant differences in age, gender, and education backgrounds among the groups (*P*>0.05, Table 1). All subjects were right-handed. Adults with NSCLP underwent correction of cleft lip surgery about 1 year before the recruitment. The aNSCLP group had AR for at least 3 months (30 min/one trial, 2-5 times/week), while the bNSCLP group had no AR. The key exclusion criteria were brain structural abnormalities, neurological or psychiatric disorders, and MRI contraindications. The Medical Research Ethics Committee of Capital Medical University (Beijing, China) approved the study protocol in accordance with the recommendations of the declaration of Helsinki for investigation of human participants. All participants provided written informed consent after being informed of the study details.

### Articulation rehabilitation

The Chinese characters included in the AR came from the Chinese language clear degree list [61], which covered all Chinese syllables, including all consonants and vowels. The goal of the rehabilitation was to achieve a score of 86 points (full credit was 100 points) in articulation test [61] for NSCLP adults; this was considered a threshold line of rehabilitation. The AR included three parts: pharyngeal closure function training, function training of articulation organ, and speech training. Pharyngeal closure function training aimed at the resonance problem of adults with NSCLP to improve their excessive nasal sound and nasal leak problems by feeling the airflow from the mouth. Function training of articulation organ focused on training of muscles and joints of lip, tongue, soft palate and other vocal auxiliary organs, to enhance their strength, extension, flexibility, and accuracy. Speech training followed “phoneme-syllable-phrase-sentence-essay conversation, increasing difficulty gradually. Pronunciation training was performed according to the Mandarin Chinese phonetic system training method, which contained three parts: 1) Phoneme training: for the organ part of pronunciation: from anterior to posterior; for the pronunciation method: from easy to difficult, aspirated-not aspirated, plosive-fricative-affricate. 2) Syllable training: According to the abnormal pronunciation of adults with NSCLP, a set of syllable training tables was developed with consonants as initials.3) Phrase training: Double-syllable words training was mostly used.

### MRI scan protocols

MRI scans were performed using a 3.0-T Siemens trio scanner vision whole-body MRI system and a 32-channel head coil (Beijing Chaoyang Integrative Medicine Emergency Medical Center, Beijing, China). The parameters of brain scan were as follows: T1-weighted structure image: repetition time=9.4ms; echo time=4.6ms; flip angle=10; acquisition matrix=240×240; voxel size =1×1×1 mm^3^; 200 contiguous axial slices.

### Structure MRI preprocessing

The MRI data were processed with the Computational Anatomy Toolbox 12 (CAT12, http://www.neuro.uni-jena.de/vbm/download/) implemented in Statistical Parametric Mapping12 (SPM12, http://www.fil.ion.ucl.ac.uk/spm). T1-weighted images were converted to NIFTI files and corrected for bias–field inhomogeneities, then spatially normalized using the DARTEL algorithm [56], and segmented into gray matter (GM), white matter (WM), and cerebrospinal fluid (CSF) [57]. The segmentation process was further enhanced by accounting for partial volume effects [58]. The CAT12 toolbox contained a topological correction performed through an approach based on spherical harmonics [59], as well as an established novel algorithm for extracting the cortical surface [60], which allowed computation of multiple morphometric parameters, including cortical thickness [60], fractal dimension [21], local curvature-based gyrification index, and absolute mean curvature (AMC) [19]. Central cortical surfaces were created for both hemispheres separately. Finally, normalized and modulated GM images were smoothed with a Gaussian kernel of 8 mm (full-width at half maximum), and all surface-based images were resampled and smoothed with a Gaussian kernel of 20 mm (full-width at half maximum).

### Statistical analysis

For demographics and behavior data, three groups were compared using SPSS 22.0 software. The categorical variables were tested by chi-square test; non-normal distribution data was expressed as median (Interquartile range, IQR), and was tested by Mann-Whitney U-test nonparametric test. *P* < 0.05 was considered statistically significant.

For imaging data, Matlab2013b, SPM12 was used to perform two-sample t-test on the GMV map of each pair between aNSCLP, bNSCLP, and NC, and total intracranial volume (TIV) was added as a covariate. To determine differences in CT, FD and gyrification between groups, two-sample t-test was performed using CAT12 and SPM12 for each pair between aNSCLP, bNSCLP, and NC, across each hemisphere. Statistical significance was defined as *P* < 0.05, cluster/peak-level FWE-corrected and a minimum cluster extent of 20 voxels/vertices.

## Supplementary Material

Supplementary Tables
