# Supplementary material for: Articulation rehabilitation induces cortical plasticity in adults with non-syndromic cleft lip and palate
Source: Aging (Albany NY). 2020 Jul 3;12(13):13147–59. doi: 10.18632/aging.103402 (PMC7377881; doi:10.18632/aging.103402)
Supplement: Supplementary Tables [file aging-12-103402-s001..pdf]

SUPPLEMENTARY TABLES

Supplementary Table 1 Brain regions with significant differences in gray matter volume between aNSCLP, bNSCLP and NC.

Brain regions	AAL Area	Peak MNI coordinates			T-values	Cluster size
		X	Y	Z		
**bNSCLP vs. NC**						
Right Inferior Frontal Gyrus	Frontal_Inf_Tri_R<br>Frontal_Mid_R	44	26	21	4.415*	322
**aNSCLP vs. bNSCLP**						
Left Postcentral Gyrus	Postcentral_L<br>Parietal_Inf_L	-32	-31	42	-4.901*	490

MNI, Montreal Neurological Institute;
A two-sample t-test was performed between each pair in aNSCLP, bNSCLP and NC. Results with * are thresholded at *P*<0.05, cluster-level FWE-corrected.
Atlas labelling was performed according to the AAL atlas [1].

Supplementary Table 2. Brain regions with significant differences in surface indexes between aNSCLP, bNSCLP and NC.

T value	Cluster size	Surface index (mean±SD)		Overlap of atlas region	
**bNSCLP vs. NC**					
cortical thickness		bNSCLP	NC		
4.8	75	2.273±0.240	2.608±0.239	65%	Parahippocampal(L)
				35%	Fusiform(L)
**aNSCLP vs. NC**					
cortical thickness		aNSCLP	NC		
4.9	197	3.459±0.152	3.243±0.137	100%	Superior frontal(R)
fractal dimension		aNSCLP	NC		
4.7	91	2.460±0.208	2.726±0.161	100%	Inferior parietal(R)
gyrification		aNSCLP	NC		
4.5	89	29.668±1.909	26.977±1.892	48%	Temporal pole(R)
				38%	Inferior temporal(R)
				14%	Entorhinal(R)
**aNSCLP vs. bNSCLP**					
gyrification		aNSCLP	bNSCLP		
4.6	126	29.472±1.438	27.374±1.273	100%	Inferior parietal(L)
4.5	96	32.299±0.950	30.905±0.943	58%	Postcentral(R)
				42%	Supramarginal(R)

A two-sample t-test was performed between each pair in aNSCLP, bNSCLP and NC. Results are thresholded at *P*<0.05, cluster-level and peak-level FWE-corrected. (L)/(R) means left/right hemisphere. Atlas labelling was performed according to the DesikanKilliany atlas [2].

Supplementary References

1. Tzourio-Mazoyer N, Landeau B, Papathanassiou D, Crivello F, Etard O, Delcroix N, Mazoyer B, Joliot M. Automated anatomical labeling of activations in SPM using a macroscopic anatomical parcellation of the MNI MRI single-subject brain. Neuroimage. 2002; 15:273–89. https://doi.org/10.1006/nimg.2001.0978. PMID:11771995

2. Desikan RS, Ségonne F, Fischl B, Quinn BT, Dickerson BC, Blacker D, Buckner RL, Dale AM, Maguire RP, Hyman BT, Albert MS, Killiany RJ. An automated labeling system for subdividing the human cerebral

cortex on MRI scans into gyral based regions of interest. Neuroimage. 2006; 31:968–80. https://doi.org/10.1016/j.neuroimage.2006.01.021 PMID:16530430
